# Efficient calculation of steady state probability distribution for stochastic biochemical reaction network

**DOI:** 10.1186/1471-2164-13-S6-S10

**Published:** 2012-10-26

**Authors:** Shahriar Karim, Gregery T Buzzard, David M Umulis

**Affiliations:** 1 Department of Agricultural and Biological Engineering, Purdue University, West Lafayette, USA; 2 Department of Electrical and Computer Engineering, Purdue University, West Lafayette, USA; 3 Department of Mathematics, Purdue University, West Lafayette, USA

## Abstract

The Steady State (SS) probability distribution is an important quantity needed to characterize the steady state behavior of many stochastic biochemical networks. In this paper, we propose an efficient and accurate approach to calculating an approximate SS probability distribution from solution of the Chemical Master Equation (CME) under the assumption of the existence of a unique deterministic SS of the system. To find the approximate solution to the CME, a truncated state-space representation is used to reduce the state-space of the system and translate it to a finite dimension. The subsequent ill-posed eigenvalue problem of a linear system for the finite state-space can be converted to a well-posed system of linear equations and solved. The proposed strategy yields efficient and accurate estimation of noise in stochastic biochemical systems. To demonstrate the approach, we applied the method to characterize the noise behavior of a set of biochemical networks of ligand-receptor interactions for Bone Morphogenetic Protein (BMP) signaling. We found that recruitment of type II receptors during the receptor oligomerization by itself doesn't not tend to lower noise in receptor signaling, but regulation by a secreted co-factor may provide a substantial improvement in signaling relative to noise. The steady state probability approximation method shortened the time necessary to calculate the probability distributions compared to earlier approaches, such as Gillespie's Stochastic Simulation Algorithm (SSA) while maintaining high accuracy.

## Introduction

Many biological networks exhibit stochasticity due to a combinatorial effect of low molecular concentrations and slow system dynamics. One important biological context where stochastic events likely have a large impact is the Bone Morphogenetic Protein (BMP) signaling pathway. BMPs make up the largest subfamily of the Transforming Growth Factor-*β *superfamily and are involved in numerous processes including growth, differentiation and diseases [1]. Due to their potency at driving development, they are also of great value for stem-cell differentiations in cell culture. BMPs activate near maximal signaling at 1*nM *concentration, have very slow binding kinetics and require oligomerization between multiple receptor subunits [1]. These properties naturally lead to conditions for significant and long-duration stochastic fluctuations in cellular signaling. Interestingly, variability of BMP signaling appears to be very low *in vivo*, while it is very high in stem cell culture studies [2]. To understand the differences between *in vivo *and *in vitro *signaling and determine how various receptor oligomerization events might alter the signal and noise, a more efficient means of solving the steady state distributions for stochastic model was needed that would allow for continuation of both parameters and levels of the BMP pathway components.

Stochastic regulation can negatively impact the robustness of the system [3,4] or instead, constructively contribute to the phenotypic variation [5-7] in a species. In stochastic reaction networks, the state of a species traverses different trajectories in a probabilistic manner and the distributions of states can be difficult to predict. As more biological data is available, stochastic modeling is becoming increasingly popular to estimate properties in networks where the time evolution of the system is unpredictable and dependent on unavoidable randomness inherent to the system. The complete solution can be calculated from a Chemical Master Equation (CME) [8-10], that is based on a Markovian approach that captures the inherent randomness of biochemical systems.

The Chemical Master Equation (CME) describes the dynamics of the probability distribution of a species of chemical reactions. Precisely, the CME captures the rate of change of probability that a system will be in state **X **at time *t *for all the species of the system. Solution of the CME is practically intractable due to the curse of dimensionality, as the state-space of the system becomes enormously large with increases in the species number and concentrations (number of states *n^N^*, for *N *→ species, *n *→ copies of each species). Moreover, the system often involves interactions between different time-scales (slow and fast reactions, frequent and infrequent transitions between states) [11], which add further complexity. Instead, numerical approaches are commonly used [12-14] to realize the CME of the stochastic system.

In the analysis of stochastic biochemical networks, steady state probability distributions for each species in the system are determined to measure variability about the deterministic steady state. The deviation around the solution contributes to stochastic noise that can be quantified by measuring the coefficient of variation $\Lambda =\frac{\sigma }{\mu }$ (the ratio between the standard deviation *σ *and the mean level of species concentration *μ*) obtained by solving the CME [9].

Frequently, Monte-carlo based simulation approaches [9,13] are used to solve stochastic problems. But, there are drawbacks in this approach for networks where the dynamics of different intermediate states of the system are unknown and continuation of several parameters is necessary to explore the system's dependency. As a screen of parameter values becomes necessary in such a scenario, the Monte-carlo based approach doesn't prove to be efficient, as it generally takes longer time to numerically simulate the process and satisfy the imposed conditions. Moreover, simulation times increase with increases in the total number of molecules, species and the number of interactions between species.

In order to ameliorate the computational cost and complexity, we present a method here to approximate the steady state probability distribution by 1) reducing the system's state-space to a finite dimension using truncated state-space method [15] and 2) subsequently, translating an eigenvalue problem associated with a CME to a system of linear equations. We illustrate that the eigenvector (for an eigenvalue = 0) that represents the steady state probability distribution can be solved algebraically by approximating it as a system of linear equations. Previously, the influence of stochastic fluctuations on system behavior was studied also in [16], where a moment closure approach was applied to reduce the complexity associated with the identification of distributions. In contrast to the previous studies, here we use a truncated state-space for steady state probability distribution approximation, which is arguably more general since we make no assumptions on the relationships of the moments of the distribution.

The usefulness of the proposed method is illustrated by considering the example networks of BMP signaling, as described earlier in [17]. Here we examine two potential mechanisms of BMP signaling: 1) regulation between the type I and type II receptors, and 2) regulation by secreted co-factors, such as Crossveinless-2 (Cv-2). First, we apply the approach to the recruitment of Type II receptor into a BMP-bound type I receptor complex to see if such step of receptor oligomerization contributes qualitatively to the noise profile of the system. Following this, we extend our earlier work that focused on extracellular regulation of BMP signaling by SBPs to evaluate the calculation approach and compare results to the Type I/Type II receptor system.

Unlike SBPs, which tend to improve the signal to noise ratio, we did not see a significant change in stochastic variability with inclusion of Type II receptors. This result supports a previous assumption made in [4] where the recruitment of Type II receptor was excluded to simplify the modeling steps while characterizing the noise profile of a SBP regulated BMP signaling system.

## Methods

### Proposed approximation method

The Chemical Master Equation (CME), which is a set of first order differential (ODE) equations, demonstrates loss and gain of probabilities of discrete states of a system [10] and is often applicable to represent the stochasticity of the system. Consider a well-stirred system at thermal equilibrium of *N *different species {*S*_1_, *S*_2_, . . . ,* S_N_*} with {*X*_1_, *X*_2_, *. . *. , *X_N_*} molecules respectively, participating in a total of *M *biochemical reactions *R_μ_*, where *μ *= 1, 2, . . . *M*. The state of such a system is represented by the copy number (*X_n_ molecules*) of each species (*S_n_*) at any given time *t *and is represented as **X **= [*X*_1_(*t*), *X*_2_(*t*), . . . , *X_N_*(*t*)]. Unless a non-zero initial state is assigned, the default initial species concentrations are always zero (*X_n_*(*t *= 0) = 0, *where *1 ≤ *n *≤ *N*).

Two other quantities are further required to construct the system: 1) a state-change vector *ν_μ _*and 2) propensity functions [8,12,14,18] for the reactions *R_μ_*, *μ *= {1, 2, . . . , *M*}. The state-change vector *ν_µ _*for reactions *R_μ _*is defined as *ν_μ _*= [*ν*_1*μ*_, *ν*_2*μ*_, . . . ,* ν_Nμ_*]*^T^*, where *ν_nμ _*represents the change in concentration of species *S_n_*, caused by the occurrence of *R_μ _*reaction of the underlying biochemical system. These equations fully define the system and the time evolution of the probability function *P*(**X**, *t*) can be obtained by the solution of the Chemical Master Equation(CME) [8,14,18]:

(1) $$\begin{array}{c} \frac{\partial P\left(X,t\right)}{\partial t}=\overset{M}{\underset{\mu =1}{\sum }}\left(a_{\mu }\left(X-\nu_{\mu }\right)P\left(X-\nu_{\mu },\;t\right)-\right)\\ \;\;\;\;\;\;\;\;\;\;\;\;\;\;\;\;\;\left(a_{\mu }\left(X\right)P\left(X,t\right)\right)\left[\left(X+v_{\mu }\right)\in \Omega \right] \end{array}$$

Here, [(**X **+*ν_μ_*) ∈ Ω] is 1 if **X **+*ν_μ _*∈ Ω and 0 otherwise. The CME representing the rate of change of probability *P*(**X**, *t*) in an in finitely large state-space **X **∈ Ω is given by taking Ω to be the non-truncated space: Ω = ℕ*^N^*, ℕ = {0, 1, 2 . . .}

In Eq.1, *a_μ _*represents the propensity function to account for transition from a given state **X** to any other state, and *ν_μ _*indicates the stoichiometry of the reaction *μ *that results in such a transition. Eq.1 is a linear system of differential equations and may be rewritten as follows:

(2) $$\frac{\text{d }P}{\text{d }t}=LP$$

where P is the probability distribution *P*(**X**, *t*) for a vector **X **= [*X*_1_, *X*_2_, . . . , *X_N_*] and L is the time independent connection operator. For the steady state (SS) distribution *P_ss_*, we have:

(3) $$\begin{array}{l} i)P_{ss}{}^{X}\geq 0;\;\;\;\forall X\in \Omega \;\;\;\;\;\;\;ii)\sum_{X\in \Omega }P_{ss}(X)=1\\ \;\;\;\;\;\;\;\;\;\;\;\;\;\;\;\;\;\;\;\;\;\;\;\;\;\;\;\;\;\;\;\;\;\;\;\;and\;\;iii)\;\;\;LP_{ss}=0, \end{array}$$

We assume that the deterministic steady state (SS) is unique. The non-truncated state-space Ω can be replaced with a truncated state-space $\hat{\Omega }$[15,19] to approximate the probability distribution *P*(**X**, *t*). We define the truncated space as:

(4) Ω^={X:αi≤Xi≤βi,∀i}

where *α_i _*and *β_i _*are extendable left and right boundaries of the truncated state-space. This approach is similar to that in [20], in which it is shown that the approximation based on the truncated space converges to the true steady state distribution as the limits of the truncated state-space converge to the limits of the original space.

The truncated state-space representation implies that given some *ε *> 0, for a sufficiently large *β_i _*> 0 and sufficiently small *α_i _*≥ 0, the steady state probability distribution *P_ss _*(**X**) is approximated to within *ε*:

$$\underset{X\in \hat{\Omega }}{\sum }P_{ss}\left(X\right)=1-\varepsilon$$

For an optimal SS probability approximation, *ε *should be made as small as possible. In the truncated state-space, Eq.3(iii) is represented as:

(5) $$\hat{L}{\hat{P}}_{ss}=0$$

where $\hat{L}$ is a matrix of propensities in $\hat{\Omega }$. To get the entries in $\hat{L}$ we use Eq.1 modified so that $P\left(X,t\right)=0\text{ if }X\;\notin \hat{\Omega }\;\text{and}\;a_{\mu }\left(X\right)\;=\;0\text{ if }X+\nu_{\mu }\notin \hat{\Omega }$. In the truncated state-space $\hat{\Omega }$, Eq.5 is an eigenvalue problem for eigenvalue *λ *= 0 and the eigenvector ${\hat{P}}_{SS}$ can be obtained algebraically, or with an iterative algorithm for a large, sparse matrix $\hat{L}$.

Instead of finding the eigenvector, which can be an ill-conditioned problem when there are nonzero eigenvalues close to 0, we translate the problem to a well-conditioned system of linear equations as follows.

We first evaluate the deterministic steady state (*Y*_0_) of the system, and then select state *X*_0_ of the discrete system closest to this deterministic steady state, where *X*_0 _= *round*(*Y*_0_). Taking ${\hat{P}}_{ss}$ to be the solution of Eq. 5 and using the fact that the deterministic steady state solution is unique, we observe that ${\hat{P}}_{ss}$ (*X*_0_) is among the largest entries of ${\hat{P}}_{ss}$. The states in $\hat{\Omega }$ are labeled as 1, 2, . . . , *K *with state *X*_0_ denoted by *j*.

Then

(6) $$\begin{array}{llll} \frac{{\hat{P}}_{ss}}{{\hat{P}}_{ss}^{j}} & ={\left[{\hat{P}}_{ss}^{1}...\;{\hat{P}}_{ss}^{j}...\;{\hat{P}}_{ss}^{K}\right]}^{T}/{\hat{P}}_{ss}^{j}\; & & \;\\ & \;={\left[{\hat{q}}_{1},...\;,{\hat{q}}_{j-1},1,{\hat{q}}_{j+1}\;...{\hat{q}}_{K}\right]}^{T}\; & & \;\\ & \; & \end{array}$$

where ${\hat{q}}_{k}=\frac{{\hat{P}}_{ss}^{k}}{{\hat{P}}_{ss}^{j}}$, *k *= 1 . . . *K*, ${\hat{q}}_{j}=1$. With $\hat{q}=\left[{\hat{q}}_{1},...,1,...\;{\hat{q}}_{K}\right]$ Eq.5 now becomes $\hat{L}\hat{q}=0$. Let ${\hat{L}}_{k}$ be the *k^th ^*column of $\hat{L}$. Expanding $\hat{L}\hat{q}$ by column and rearranging gives the following well-conditioned problem:

(7) $$\begin{array}{c} \overset{K}{\underset{k=1,k\neq j}{\sum }}{\hat{L}}_{k}{\hat{q}}_{k}=-{\hat{L}}_{j}\;or,\\ \;\;\;\;\;\;\;\;\;\;\;\;\;\;\;{\hat{L}}^{\prime }{\hat{q}}^{\prime }=-{\hat{L}}_{j} \end{array}$$

In Eq.7, ${\hat{L}}^{\prime }$ is the matrix $\hat{L}$ with column *j *removed and ${\hat{q}}^{\prime }$is $\hat{q}$ with entry ${\hat{q}}_{j}$ removed. The error criterion for the system is checked for the calculation of ${\hat{P}}_{ss}$ until a satisfactory value is obtained (see algorithm 1 for further details).

### Application

In order to demonstrate the usability of the proposed steady state probability approximation method, we present here two example networks (example network 1 and 2) from Bone Morphogenetic Protein(BMP) mediated signaling, and characterize the stochastic behavior of the systems. In the example network 1, we consider the role of a specific extracellular protein, Crossveineless-2(Cv-2), which is part of a class of proteins known as Surface-associated BMP-binding Proteins (SBPs) [4]. Cv-2 has the ability to regulate the stochastic noise in BMP signaling, and in this example we demonstrate that the role of Cv-2 is heavily dependent on reaction kinetics of the network: for some sets of parameter values, Cv-2 increases the coefficient of variation of the steady state signaling distribution, while for other parameter values it decreases the coefficient of variation.

In the second example network, we consider a model simplification strategy as used in [4,17]. This strategy is to omit a Type II receptor recruitment step from the receptor oligomerization in a BMP patterning model, under the assumption that the simplification step does not affect the outcome of a BMP-mediated patterning model. The obtained results by the use of steady state probability approximation method provide a numerical justification for the aforementioned simplification.

### Background

During embryonic development, positional information is transduced by morphogens to underlying cells that respond to the concentration gradient of morphogen and eventually differentiate into distinct cell types [21]. For example, Decapentaplegic (*Dpp*), a drosophila homologue of BMP2/4, forms a spatial profile to pattern dorsal tissues in *Drosophila *development [21]. In a canonical BMP signaling pathway, dimeric ligands bind to receptors and form a heterotetrameric complex that consists of two Type I and two Type II receptors. The heterotetrameric receptor complex then phosphorylates the intracellular signal transducer Smad and the phosphorylated Smad forms a complex with a co-Smad. Subsequently, the Smad/Co-Smad complex accumulates in the nucleus and regulates gene expressions in a concentration dependent manner [17,22].

BMP regulation occurs at many points along the pathway, and a lot of focus has been on identifying and understanding how the ligand activity is regulated in the extracellular environment by secreted binding proteins. These include molecules such as Cv-2, HSPGs, among other reviewed in [1]. A focus of this work is to gain a better understanding of how regulation in the extracellular region impacts cell signaling noise, and eventually cell-to-cell variability.

### Example networks

**Figure 1:**
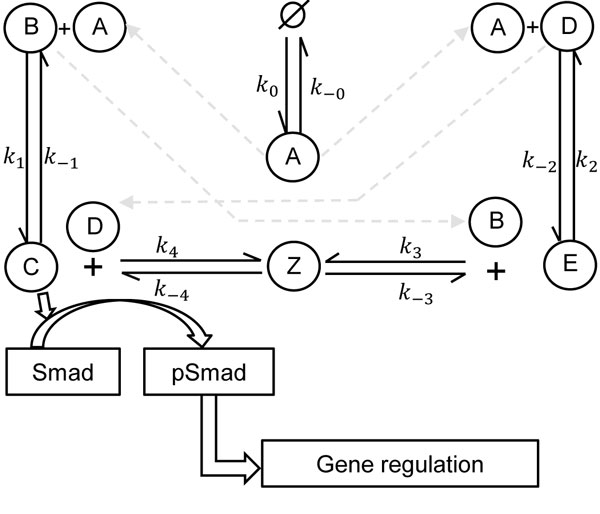
**Example network of BMP signaling**. BMP signaling is mediated by BMP:Type I Receptor (C) forms either by direct interaction between BMP (A) and Type I (B) or via an intermediate state with BMP:Type I:Cv-2 (Z). Initially B interacts with A (Type I receptor), D (Cv-2) and forms C (BMP:Type I receptor) and E (BMP:Cv-2) complexes respectively. Then the complexes C, E can generate the intermediate state BMP:Cv-2:Type I complex (node Z) by interacting with D and B respectively. In the network, only BR(node C) has the ability to turn on downstream signaling. Upon BR formation, the complex recruits type II receptor, and later initiates the phosphorylation of intracellular Smad protein. Signaling leads to pSmad accumulation within the nucleus and gene expressions of BMP targets.

In many biochemical networks, where dynamics of the intermediate interactions of different species (proteins) and molecular complexes are largely unknown, screening plays a significant role in the classification of dynamics-dependent network behavior. For example:

1. In a biochemical network where a class of secreted, surface-associated BMP binding proteins (SBPs) such as, Crossveinless-2 (Cv-2, node D as in Figure 1) [4] is allowed to regulate BMP signaling, the intermediate dynamics of the system that result in the formation and decoupling of a transient state BMP:Type I:Cv-2 (node M as in Figure 1) are largely unknown.

2. In the patterning modeling of BMP signaling pathways, it is often argued as a simplification strategy that omitting the step of recruitment of a Type II receptor to a bound BMP:Type I receptor complex doesn't affect the outcomes of patterning models [4,17,23,24]. While valid in the deterministic sense, it is not clear how this reduction impacts our estimates for noise in the sytem.

In these systems, we apply our SS probability approximation method to evaluate the probability distribution of different species and calculate the mean (*μ*), standard deviation (*σ*) and the coefficient of variation ($\Lambda =\frac{\sigma }{\mu }$; defined as the ratio between the standard deviation and the mean of any species) of the species distribution. Together with this information, we can screen the network for largely unknown dynamics of the intermediate interactions and classify solutions according to a model's ability to meet specific performance objectives.

### BMP-signaling regulation by SBPs

#### Signaling network

The single-cell local stochastic model that includes extracellular BMP(A), receptors (B), and SBPs such as, Cv-2 (D) with biochemical interactions, rate parameters, and connectivity is based on the network shown in Figure 1. Mass balance equations are listed below:

$$\begin{array}{c} R_{1}:\emptyset \overset{k_{0}}{\underset{}{\to }}A\;R_{2}:A\overset{k_{-0}}{\underset{}{\to }}\emptyset \;R_{3}:A+B\overset{k_{1}}{\underset{}{\to }}C\\ R_{4}:C\overset{k_{-1}}{\underset{}{\to }}A+B\;R_{5}:A+D\overset{k_{2}}{\underset{}{\to }}E\\ R_{6}:E\overset{k_{-2}}{\underset{}{\to }}A+D\;R_{7}:B+E\overset{k_{3}}{\underset{}{\to }}Z\\ R_{8}:Z\overset{k_{-3}}{\underset{}{\to }}B+E\;R_{9}:C+D\overset{k_{4}}{\underset{}{\to }}Z\\ R_{10}:Z\overset{k_{-4}}{\underset{}{\to }}C+D \end{array}$$

Out of all complexes (C = BMP:Type I Receptor = BR, E = BMP:Cv-2, Z = BMP:Type I receptor: Cv-2), available experimental evidence suggests that only ligand-bound receptors C (BMP:Type I Receptor = BR) initiate signaling to regulate downstream gene expression [17]. To focus on the noise compensation by regulation of receptors by SBPs, the extracellular level of BMP (A) is treated as a parameter $A=\frac{k_{0}}{k_{-0}}$ and the interactions (1-10) are simplified accordingly. For example, in the simplified model reaction *R*_3_ is represented $R_{1}^{\prime }:B\overset{Ak_{1}}{\underset{}{\to }}C$.

The simplified model as obtained from reactions *R*_1_ to *R*_10_, has 5 species {*S*_1_, *S*_2_, . . . , *S*_5_} and is described completely by a total of 8 different chemical reactions. Time evolution of all species quantities are specified by a state vector **X(t) **= [*X*_1_(*t*), *X*_2_(*t*), . . . , *X*_5_(*t*)]*^T ^*and state-change vector *v_μ _*(*μ *= 1, 2, . . . 8), corresponding to all reactions that describe the system. For example, when *μ *= 1, *v*_1_ is [-1 +1 0 0 0]*^T ^*for reaction $R_{1}^{\prime }$ of the simplified system. In this example network, techniques like those in [25] were used to verify that there is a unique steady state equilibrium and this ensures the applicability of the algorithm for this example network. Numerically, we used the polynomial root finding package hom4ps2 to ensure that there was only one equilibrium in the positive orthant [26]. It's worthwhile to mention that a similar approach is adopted in example network 2 to ensure the unique deterministic steady state. For both the networks, we numerically determined the deterministic steady state value *Y*_0_ using Newton's method as incorporated in SBTOOLBOX2 [27]. A generalized algorithm for simulation according to the steady state approximation as outlined in Methods section is given in algorithm 1.

**Algorithm 1 **Evaluate steady state (SS) distribution: $\hat{L}{\hat{P}}_{ss}=0$

**Require: **Unique deterministic SS solution *X*_0_

1. Reaction Networks with *N *Reaction *R*_1_, . . . , *R_N_*

2. Choose: *ε, γ*_0_, *γ*

3. Solve: ODE for steady state(SS) = *Y*_0_ and find discrete state *X*_0_ closest to *Y*_0_, where *X*_0_ = *round*(*Y*_0_).

4. Initiate, *α_i_, β_i_*; where $\alpha_{i}={\left(X_{0}\right)}_{i}-\gamma_{0}$, $\beta_{i}={\left(X_{0}\right)}_{i}+\gamma_{0}$

5. Determine: Truncated state-space $\hat{\Omega }$ as shown in Eq.4 and $\hat{L}$ as described after Eq.5

6. Determine: Column j of $\hat{L}$ corresponding to *X*_0_

7. Form ${\hat{L}}^{\prime }$ and ${\hat{L}}_{j}$ as describe after Eq. 7.

8. Solve: ${\hat{L}}^{\prime }{\hat{q}}^{\prime }=-{\hat{L}}_{j}$

9. Find ${\hat{P}}_{ss}=\frac{{\left[{\hat{q}}_{1},...,{\hat{q}}_{j-1},1,{\hat{q}}_{q+1}...{\hat{q}}_{K}\right]}^{T}}{\eta }$, where ${\hat{q}}^{\prime }={[{\hat{q}}_{1},...,{\hat{q}}_{j-1},{\hat{q}}_{j+1},...{\hat{q}}_{K}]}^{T}$ and *η *> 0 and $\eta =1+\overset{K}{\underset{l=1,l\neq j}{\sum }}{\hat{q}}_{l}$ is chosen so that $\underset{X\in \hat{\Omega }}{\sum }{\hat{P}}_{ss}\left(X\right)=1$

10. Verify:

**if **$\underset{X\in \hat{\Omega },X_{i}=\;\delta_{i}}{\sum }{\hat{P}}_{ss}\left(X\right)\geq \varepsilon$, for *δ_i _*= *α_i_*, or *δ_i _*= *β_i _***then**

α_i ← αi _- γ

*β_i _*_← _*β_i _+ γ*

Return to 5

end if

In the algorithm, the values of *γ*_0_, *γ *are problem dependent based on the anticipated spread of the steady state (SS) distribution. Larger *γ *and *γ*_0_ favor a larger $\hat{\Omega }$, with correspondingly better accuracy, but this comes at the expense of a larger state-space and more time required to solve the Eq.7. The parameter *ε *also controls the accuracy of the solution. In the truncated state-space, the tail of the distribution is essentially pushed in to the main part of the distribution and smaller *ε *means that less of the tail is changed.

#### Simulation and discussion

The binding kinetics between BMPs (species A, Figure 1) + receptors (species B), and BMPs + Cv-2 (species C) are largely known from the biacore analysis data [28,29]. However, the kinetic data associated with the intermediate tripartite complex BMP:Cv-2:Type I receptor (species Z, Figure 1) are currently unknown. In order to better understand the dependence of the steady state distribution on the kinetic parameters, we performed a parameter screen for the forward and reverse reaction rates (*k*_±*s*_, *s *= 3, 4) for the formation and decoupling of species Z. For each of these four parameters, we use 5 evenly-spaced points on a logarithmic scale with the range [10^-1 ^*to *10^1^] *nM*^-1^*s*^-1^ for the forward rates and [10^-3 ^*to *10^0^] *s*^-1^ for the reverse reaction rates. This produces a parameter grid that contains a total of 625 different parameter vectors.

For an appropriate comparison of the noise attenuation both in the presence and in the absence of *Cv *- 2, species C (BMP: Type I Receptor = BR) concentration should remain the same regardless of the intermediate dynamics. During simulation, the amount of available receptors (B) was fixed at 100, and a maximum of 30% receptor occupancy was allowed for the screening of the network. To ensure an equal amount of BR formation for each parameter vector, we modified the level of free ligand (A). For computational tractability, the screen is limited to a maximum continuation of 200 Cv-2 molecules, which allowed us to capture responses for all 625 different parameter sets.

**Figure 2:**
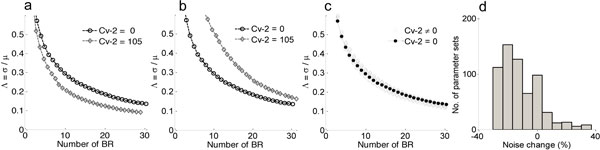
**Screening result**. **a, b, c**) Coefficient of variation $\left(\Lambda =\frac{\sigma }{\mu }\right)$ for BR (C) (1 to 30 *molecules*) formation is shown both in the presence of *Cv *- 2 = 105 *molecules *(gray) and in the absence of Cv-2 (black), where a, b, c represents the noise attenuation, noise amplification and biphasic subclasses of Cv-2 behavior respectively. **d) **Histogram of noise change for all 625 parameter sets demonstrates that the majority of solutions result in noise attenuation.

In order to quantify noise in the system we measured the coefficient of variation $\left(\Lambda =\frac{\sigma }{\mu }\right)$ that relates the standard deviation (*σ*) to the mean (*μ*) level of bound receptors. The parameter screen on the intermediate dynamics yields three primary qualitative subclasses for Cv-2 behavior in regulation of extracellular BR (C) fluctuation amplitude: i) reduced amplitude ii) increased amplitude and iii) mixed amplitude behavior [4]. Three primary types of responses of Cv-2 action on BMP fluctuations are shown in Figure 2a-c. As seen from Figure 2a, Cv-2 leads to a reduction of BR noise amplitude, and this is true for all Cv-2 ∈ [0,200]. The value of the coefficient of variation (Λ) decreases for both increases in the level of bound receptor and the level of Cv-2. The subclass of increased amplitude demonstrates that increasing the level of Cv-2 in the system increases the value of the coefficient of variation (Λ*_Cv_*_2 ≠ 0 _> Λ*_Cv_*_2 = 0_) and is valid for the range of Cv-2 values considered in the screen (for a detailed discussion on this, interested readers can refer [4]). Lastly, mixed amplitude is classified as type iii, which demonstrates that Cv-2 can both increase and decrease the level of stochastic noise in the system (Figure 2c).

To clarify Cv-2 action further, we calculated percentage change in the amplitude using $\left(\%\;\text{noise}\;\text{change}\;\text{ = }\frac{\left(\Lambda_{Cv-2=105}-\Lambda_{Cv-2=0}\right)}{\Lambda_{Cv-2=0}}\times 100\right)$ for each parameter set output for a given amount of BR production (30 complexes) and Cv-2 value (105 molecules). Based on the percent change of the coefficient of variation, we classify the screening outcome: negative percent change corresponds to noise attenuation whereas the positive change gives noise amplification. A histogram of all 625 parameters are shown in Figure 2d and in [4]. The implication of the screening result is that Cv-2 clearly reduces the variability of receptor activation throughout the range of Cv-2 tested. However, as demonstrated in Figure 2d, such a phenomenon as exhibited by SBPs like Cv-2 is found to be highly parameter dependent.

During the simulation, the kinetic rate constants for the intermediate complex BMP:receptor:Cv-2 (node Z, Figure 1) formation and decoupling were chosen from the parameter grid and representative kinetic rate constants for three different type of Cv-2 responses are enumerated in Table 1.

**Table 1:** Kinetic rates, Figure 2(a,b,c)

Figure	k_3 _(molecule^-1 ^sec^-1^)	k_-3_ (sec^-1^)	k_4_ (molecule^-1 ^sec^-1^)	k_-4_ (sec^-1^)
2a	1.3282	0.0100	1.3282	0.0100
2b	0.0133	1.0000	0.1328	0.0100
2c	0.1328	1.0000	0.4200	1.0000

#### Analysis of Type II receptor recruitment process

**Figure 3:**
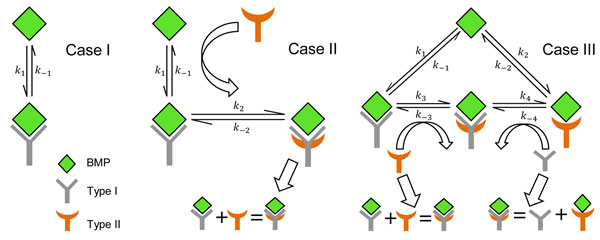
**Network cases for Type II recruitment analysis in context of Drosophila melanogaster development**. **Case I) **Recruitment of Type II is overlooked here and it imitates the simplified model used in previous studies. In this type of network, BMP:Type I complex (B*R*_1_) acts as the sole signaling complex. **Case II) **Upon the formation of a BMP: Type I complex, subsequent recruitment of Type II receptor is considered here. But a direct interaction between BMP and Type II receptor doesn't happen in the network. Here, a tripartite complex BMP:Type I:Type II (B*R*_1_*R*_2_) activates the downstream pathways. **Case III) **Similar to Case II, but with an exception that a direct interaction between BMP and Type II receptor is allowed to form a BMP:Type II complex (*BR*_2_) by changing *BR*_1_ to *BR*_2_. The kinetic equations are equivalent to the SBP system investigated in [4].

In the signaling network shown in Figure 3, recruitment of Type II (= *R*_1_) receptors can happen in two different ways: 1) BMP binds with Type I (=* R*_1_) first and subsequently, recruits Type II receptors to form a tripartite complex BMP:Type I: Type II (*BR*_1_*R*_2_), and 2) BMP directly interacts with Type I and Type II separately, and an intermediate state forms a tripartite BMP:Type I: Type II complex. In both situations, BMP:Type I:Type II complex (B*R*_1_*R*_2_) is the sole signaling complex responsible for the activation of downstream pathways.

All possible biochemical interactions that represent the ligand binding with Type I receptors and further recruitment of Type II receptors are:

$$\begin{array}{c} r_{1}:B+R_{1}\overset{k_{1}}{\underset{}{\to }}BR_{1}\;r_{2}:BR_{1}\overset{k_{-1}}{\underset{}{\to }}B+R_{1}\;r_{3}:B+R_{2}\overset{k_{2}}{\underset{}{\to }}BR_{2}\\ r_{4}:BR_{2}\overset{k_{-2}}{\underset{}{\to }}B+R_{2}\;r_{5}:BR_{1}+R_{2}\overset{k_{3}}{\underset{}{\to }}BR_{1}R_{2}\;r_{6}:BR_{1}R_{2}\overset{k_{-3}}{\underset{}{\to }}BR_{1}+R_{2}\\ r_{7}:BR_{2}+R_{1}\overset{k_{4}}{\underset{}{\to }}BR_{1}R_{2}\;r_{8}:BR_{1}R_{2}\overset{k_{-4}}{\underset{}{\to }}BR_{2}+R_{1} \end{array}$$

The chemical interaction of Case II can easily be obtained from the interactions (*r*_1_ to *r*_8_) of Case III (Figure 3) by equaling the kinetic rate constants *k*_±2_ and *k*_±4_ of Case III to zero. For the kinetics, we relied on the published data [1]. The rate at which a Type II receptor is recruited upon formation of a BMP:Type I complex (B*R*_1_) is comparatively faster than the rate of BMPs and Type I receptors interactions [17]. However, exact values of the rates of formulation of (*BR*_1_*R*_2_) complex are not readily available, and hence, parameters were screened over the physiological ranges with values between [10^-1 ^*to *10^1^] *nM*^-1 ^*s*^-1^ for the forward rates and [10^-3 ^*to *10^0^] *s*^-1^ for the reverse reaction rates.

#### Simulation and discussion

To simulate the networks (as shown in Figure 3) for the calculation of the coefficient of variation Λ, we applied the truncated state-space approximation. During the simulation, a target of 1 to 30 signaling complexes (*BR*_1_ for Case I and *BR*_1_*R*_2_ for Case II, Case III) in the extracellular region is considered so a direct comparison can be made for the coefficient of variation $\left(\Lambda =\frac{\sigma }{\mu }\right)$ between B*R*_1_ and B*R*_1_*R*_2_.

**Figure 4:**
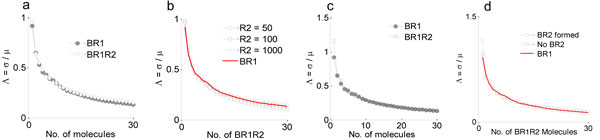
**Comparison of Λ**. **a) **The coefficient of variation of *BR*_1_ (calculated from Case I Figure 3)and *BR*_1_*R*_2_ complexes (calculated from Case II Figure 3) is compared. The variability of the system seems to be invariant in the presence of Type II. **b) **Concentration dependency of Λ as a function of *R*_2_. **c) **Same as plot "a", however, direct interaction of BMP and Type II is allowed as in Case III, Figure 3. It's clear that the stochasticity of the system does not change over the range of values tested. **d) **Summary of *BR*_1_*R*_2_ formation and its impact on signaling noise.

The coefficient of variation (Λ) for *BR*_1_*R*_2_ remains very close to the coefficient of variation of *BR*_1_ as shown in Figure 4a. Proximity in the coefficient of variation between *BR*_1_ and *BR*_1_*R*_2_ (as shown in Figure 4a) demonstrates that the stochastic variability of the system is not affected by the recruitment of the Type II receptor. It is also found that increasing the concentration of *R*_2_ brings the coefficient of variation of *BR*_1_*R*_2_ into very close agreement with the coefficient of variation of *BR*_1_ as shown in Figure 4b. A similar outcome is obtained from the simulation of Case III of the Figure 3 and the result is shown in Figure 4c. Finally, all the outcomes are summarized in Figure 4d, where it is shown that regardless of the different cases as shown in Figure 3 the coefficient of variation (Λ) of *BR*_1_*R*_2_ is approximately equal to that of *BR*_1_.

**Figure 5:**
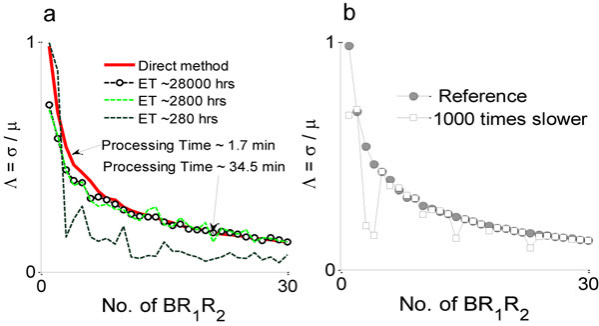
**Comparison between SSA and Direct SS method**. **a) **In Gillespie's method larger 'End Time' (ET) is required (which translates into a higher processing cost and time) to ensure the accuracy of outcome. Three different ET: 280 *hrs*, 2800 *hrs*, 28000 *hrs *are shown. **b) **Effect of kinetics associated with *BR*_1_ interacting with *R*_2_. The steps of interactions are clearly shown in Case II of Figure 3.

Additionally, it is also found from the simulated result that the rate at which the BMP:Type I recruits Type II receptor (Case II in Figure 3) also decides the effect of Type II recruitment process on the stochastic variability of the system. With a comparatively slower rate, the coefficient of variation tends to oscillate as observed in Figure 5b. When the recruitment rate is slower than the formation rate of BMP:Type I complex, free Type II receptors fail to get frequent access to BMPs via the BMP:Type I:Type II tripartite complex, and can cause the concentration of BMP:Type I:Type II complex to oscillate more than the case with a comparatively faster dynamics. Thus, mitigating noise is not a natural output of receptor oligomerization + transudction and instead, requires another co-factor such as Cv-2 [4].

### Benchmarking of Direct SS approximation method

**Figure 6:**
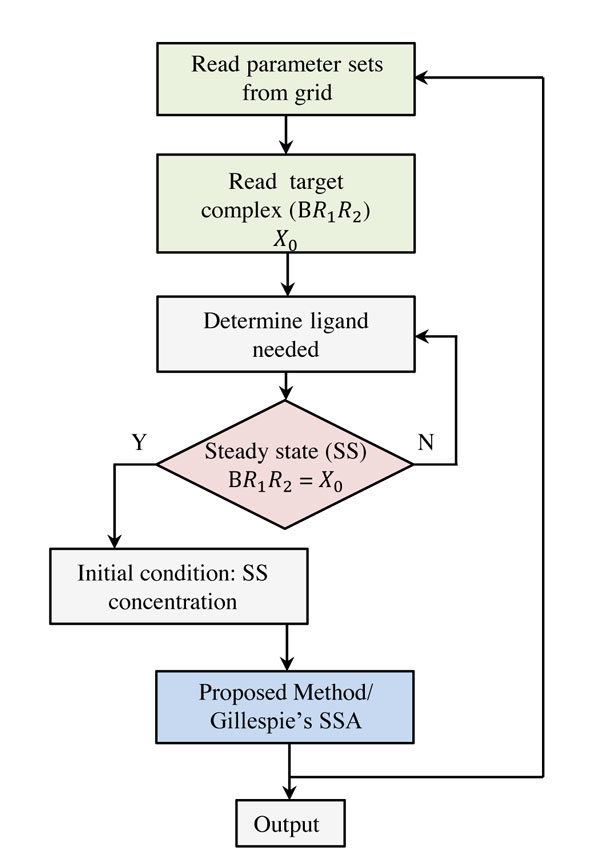
**Benchmarking of Direct SS approximation method**. Benchmarking of Direct SS approximation method.

Carrying out large-scale stochastic simulation can be time consuming but calculation of the approximate solution via a truncated state-space can greatly improve the speed. In order to show the performance improvement in terms of computational cost and time of direct SS approximation in the analysis of stochastic biochemical networks, we benchmarked the method by comparing it with Gillespie's stochastic simulation algorithm (SSA) method [9] for numerical calculations of stochastic biochemical networks. In the benchmarking, the processing time taken by each method was calculated based on the steps in the blue box as mentioned in the flow chart diagram (Figure 6). The sample problem was calculated for both methods on the same hardware and software configuration: *Processor*: Intel(R) Xeon (R) CPU E5405, 2.00 GHz (quad-core), *RAM*: 16 GB, SBTOOLBOX2 [27] and Matlab R2010a with SiMBiology 3.0.

The processing time and computed Λ values for a target *BR*_1_*R*_2_ = 20 for Case II, Figure 3, is provided in Table 2 to show the accuracy and time gain that can be obtained if the proposed direct SS distribution approximation method is used. Gillespie's SSA takes longer to generate an output that contains enough information to calculate the distribution as compared to the time taken by Direct SS approximation method. The problem becomes severe when continuation of a multiple parameters are necessary to explore the system's parameter dependency as done previously in [4].

**Table 2:** Benchmarking: Gillespie's SSA and Direct SS approximation for a target ***BR*_1_*R*_2_ = 20**

Method	End Time (ET) in Gillespie's SSA(hrs)	Processing Time (sec)	Λ
Direct SS approximation	Not Applicable	0.4 -0.6	0.1707

Gillespie's SSA	28000	90-95	0.1705
	2800	8-10	0.1878
	1390	4-5	0.2254

In Table 2, the term 'End time (ET) in Gillespie's SSA' corresponds to the amount of time the system dynamics were allowed to evolve. The accuracy of the Gillespie's SSA approach depends on the 'End time in Gillespie's SSA'(directly contributes to the processing time) set in the model simulation, and is shown clearly in Figure 5a and Table 2. Very low propensities require long simulation times in Gillespie's SSA due to the infrequency of events. Accuracy of Gillespie's method for the sample example increases as the 'End time in Gillespie's SSA' is increased. This large simulation time in turn directly impacts the processing time, resulting in a large computational cost to achieve the desired accuracy (Table 2).

## Conclusions

In this study, we illustrate an approach of determining the steady state probability distribution efficiently to carry on continuation in multiple variables within a large-scale parameter screen. The approach is demonstrated further with a couple of applications, where we investigated 1) the dynamic dependency of a class of proteins, known as SBPs, in the regulation of BMP signaling, and 2) the binding of Type II receptor in BMP signaling. The results suggest that the recruitment of a type II receptor in BMP signaling doesn't affect the stochasticity of the system over the range of concentration and parameters investigated. Direct calculation of the SS probability distribution can be successfully applied to systems with a unique deterministic SS solution, and future work will investigate similar approaches for other biochemical systems.

## Competing interests

The authors declare that they have no competing interests.

## Authors' contributions

MSK designed the research, contributed to algorithm development, performed simulation and data analysis, and wrote the manuscript. GB contributed to algorithm development, discussed the results and wrote the manuscript. DU contributed in research design, discussed the results and wrote the paper. All authors contributed to replying to reviewers' comments and approved the final manuscript of the paper.
